# Politicians lie, so do I

**DOI:** 10.1007/s00426-017-0954-7

**Published:** 2017-11-30

**Authors:** Jérémy Celse, Kirk Chang

**Affiliations:** 1 Behavioral Economics, Department of Management, Organization & Entrepreneurship, Burgundy School of Business, 29 rue Sambin, BP 50608, 21006 Dijon Cedex, France; 2 Organizational Behavior & Research Convenor, Salford Business School, University of Salford, The Crescent, Salford, Manchester, M5 4WT UK

**Keywords:** Dishonesty, Leadership, Lying, Politicians, Priming, Wellbeing

## Abstract

This research analyzed whether political leaders make people lie via priming experiments. Priming is a non-conscious and implicit memory effect in which exposure to one stimulus affects the response to another. Following priming theories, we proposed an innovative concept that people who perceive leaders to be dishonest (such as liars) are likely to lie themselves. We designed three experiments to analyze and critically discussed the potential influence of prime effect on lying behavior, through the prime effect of French political leaders (including general politicians, presidents and parties). Experiment 1 discovered that participants with non-politician-prime were less likely to lie (compared to politician-prime). Experiment 2A discovered that, compared to Hollande-prime, Sarkozy-prime led to lying behavior both in gravity (i.e., bigger lies) and frequency (i.e., lying more frequently). Experiment 2B discovered that Republicans-prime yielded an impact on more lying behavior, and Sarkozy-prime made such impact even stronger. Overall, the research findings suggest that lying can be triggered by external influencers such as leaders, presidents and politicians in the organizations. Our findings have provided valuable insights into organizational leaders and managers in their personnel management practice, especially in the intervention of lying behavior. Our findings also have offered new insights to explain non-conscious lying behavior.

## Introduction

Lying is one of the most controversial abilities that humans possess. On the one hand, people generally dislike being lied to and are keen to interrogate why liars lie. On the other hand, people may engage in lying behavior if they believe it is necessary and worthy to do so. For instance, scholars have discovered that people generally want to maintain a positive image about themselves and feel good about themselves when looking into the mirror, and one of the expedient ways to achieve a positive image is lying, e.g., saying something not true but still feeling good (*ethical dissonance*; Ayal and Gino 2011; Barkan, Ayal, & Ariely, 2015). Interestingly, although virtually all societies have sanction or penalty against lying behavior, deception and the ability of lying are still essential for polite interaction and self-preservation (Serota, Levine & Boster, 2010).

To analyze the motives and mechanism underlying lying, scholars have examined the lying behavior through a variety of perspectives. These perspectives include, for instance, development of lying ability (Evans and Lee 2013), intention and justification of lying (Bok 1999), ubiquity of lying (DePaulo 2004), face-to-face vs. computer-mediated lying (Hancock, Thom-Santelli, & Ritchie, 2004), lying detection ability (Levine, Serota, & Shulman, 2010) and emotional inhibitors of lying (Celse, Chang, Max, & Quinton, 2016). Lying brings interests to the person who lie (liar), either immediately or at a later stage (DePaulo 2004). Liars are evaluative and critical about their pay-off of lying, as Becker (1968) claim that liars calculate the benefits and risks of lying beforehand but only when the *pay-off* is satisfactory can lying behavior emerge. A common thread across the aforementioned studies is: lying is a conscious behavior. Liars lie because they want to lie.

Interestingly, however, scholars also suggest that lying includes both conscious and unconscious components (Hochman, Glockner, Fiedler, & Ayal, 2015). Scholars have well studied the phenomenon of conscious lying and provided valuable insights into the mechanism of lying behavior (e.g., Bok 1999; Evans and Lee 2013), but unconscious lying seems to draw limited attention [see exception in Gino (2015) research, which discovers that people who value morality may still cheat]. Gino’s finding is meaningful as it implies a possibility that liars may not be aware of their own lying behavior. Specifically, scholars have discovered that individual differences are critical to the lying rates and their propensity to lie (Serota et al., 2010). Bond and DePaulo (2008) report substantial variation in both people’s demeanor and people’s ability to lie. Kashy and DePaulo (1996) claim that people’s ability to lie successfully may impact how often they lie. Celse et al. (2016) suggest that envy refrains lying behavior. Taken together, prior studies have shown some insights into the view that lying may occur in the absence of conscious intervention.

Following this line of research, our project therefore aims to examine the possibility of prime-triggered lying. Prime is a non-conscious and implicit memory effect in which exposure to one stimulus affects the response to another. Inspired by the prime theories, we propose an innovative hypothesis that people who perceive politicians to be liars are likely to lie themselves. Thus our research aims to examine the link between politician-prime and lying behavior, thereby contributing to knowledge of non-conscious lying and will also provide suggestions for the intervention of lying behavior.

## Theoretical background

Human behaviors are broadly divided into three categories: *consciousness driven, non-consciousness driven*, or a *combination* of the two aforementioned. Consciousness-driven behaviors are resource demanding, as people need to mobilize their cognitive resources and these processes are often related with individual intentions and controllability, i.e., conscious processes tend to operate with individual awareness and efforts (Bargh and Chartrand 2000). Non-consciousness-driven behaviors are automatic, unintentional and hence relatively effortless, i.e., non-conscious processes tend to operate outside individual awareness (Bargh and Williams 2007). Following this logic, lying is viewed as a consciousness-driven behavior, as lying is related with individual intention and preparation behind it (Becker 1968; Kashy and DePaulo 1996). This view is echoed by Gino and Mogliner (2014). They indicate that, compared to time, contextual factors such as money and power are more likely to increase unethical behavior.

Lying could be intervened by conscious inhibitors such as corporate ethics, work ethos, honour codes and emotions (Celse et al., 2016; Mazar, Amir, & Ariely, 2008). A group of scholars also indicate that moral reminders (e.g., religious statements, honor codes) reduce participants’ tendency to lie, by which moral reminders act as a moderator in decreasing the frequency of dishonest behavior (Ayal, Gino, Barkan, & Ariely, 2015; Shu, Mazar, Gino, Ariely, & Bazerman, 2012). Having said this, however, our views are different from hose of prior studies. We argue that lying may not necessarily occur at the consciousness level, as priming studies have provided preliminary support to the possibility of lying without individual awareness. To examine the argument above and the rationale behind, this paper now turns to analyze the concept of prime and discuss the prime–lying nexus, respectively.

### Prime and priming effect

In layman’s term, priming is a non-conscious memory effect, in which exposure to one stimulus affects the response to another. The construct of priming is crafted by Meyer and Schvaneveldt (1971), indicating that human behavior can be initiated automatically by presenting relevant situational cues with the mediation of non-conscious perceptual or evaluative processes. Bargh, Chen and Burrows (1996) continue this line of research and suggest that when people behave in the same way when exposed to a particular situational cue, their behavioral response soon becomes associated with the situational cue automatically. Through repeated events, the mere presentation of the situational cue is sufficient to trigger the corresponding behavior automatically, and the situational cue is then described as a *prime* (Bargh 1996).

Bargh and Chartrand (2000) define priming as passive, subtle and unobtrusive activation of relevant mental representations by external environmental stimuli, including exposure to semantic concepts, short messages, visual images and physical sensations. Priming activates temporarily an individual’s mental representation and is concerned with examining how “these internal readinesses interact with environmental information to produce perceptions, evaluations … motivations and behavior (p. 258)”. That is, priming is like a process of activating specific knowledge structures incidentally or unobtrusively, which then influences subsequent behavior, opinion and judgment outside individual awareness. LeBoeuf, Shafir, and Bayuk (2010) have also discovered that priming could be adopted as a technique to activate people’s mental representations and to examine how these representations affect behaviors and attitudes.

Following this logic, we argue that the activation of priming is beyond individual consciousness and priming effect may lead to behavior in the absence of conscious intervention. Our argument may sound bold, but it could be further justified in several ways. First, priming effect can be manifested via the principle of ideomotor action (PIA: James 1890): simply thinking about an action is sufficient to drive the appearance of that movement, unless the person consciously engages in efforts to avoid such appearance. A good illustration of PIA is supplied by Knuf, Aschersleben and Prinz (2001). Their study has found that when watching sport video clips, participants tend to perform the movements they see (perceptual induction) or perform movements suited to achieve what they would like to see (intentional induction). When watching a ball traveling toward a target, participants may also move their limbs and tense their muscles in accordance to the movement in the video clips.

Second, priming effect can be clarified via imitation effect, i.e., imitation cues may postulate an automatic link between perception and behavior; more specifically, perception of certain behaviors and actions can lead to the performance of those actions (Bock 1986; Smeets and Brenner 1995). A good illustration of imitation effect is available from Berkowitz’s experiment, by which aggressive primes are found to affect subsequent aggressive behavior. Berkowitz (1984) devises a two-stage experiment. At Stage 1, participants are primed with the concept of hostility (or neutrality in the control condition). At Stage 2, participants are asked to punish their partner with electrical shock if their partner gives wrong answers (for the sake of research ethics, this partner is a virtual computer-acted confederate, rather than a real person). Berkowitz has discovered that participants with hostility-priming have demonstrated longer shocks to the partners than those with neutrality-priming.

Third, we have conducted literature reviews on priming and scrutinized its potential influence, but the findings of priming effect seem mixed, covering different dimensions of human cognition and attitude. For instance, a group of researchers indicate that priming effect is linked with intellectual performance (Dijksterhuis and van Knippenberg 1998), memory (Yoon et al. 2000) and conformity (Epley and Gilovich 1999), whereas other scholars suggest that priming is found to trigger the attitude of pro-social behavior (Abbate, Ruggieri, & Boca, 2013), aggression (Bargh et al. 1996), competition (Kay, Wheeler, Bargh, & Ross, 2004) and dishonesty (Cohn, Fehr, & Maréchal, 2014).

To sum, although scholars have discovered different outcomes of priming effects, one thing that they jointly agree is that activation of priming is beyond individual consciousness. Although studies of PIA and imitation effect differ in construct, they have provided preliminary credence to support our argument that the activation of priming may occur in the absence of conscious intervention and priming effect may lead to behavior, i.e., the prime–behavior nexus exists.

### Prime and lying nexus: the case of politician-priming

Following the discussion above, we hereby adopt a more critical perspective to examine the prime–behavior nexus, aiming to evaluate whether such nexus is applicable to lying behavior. Our questions are: if the answer is affirmative, how primes trigger lying behavior? What might be the mechanism behind it? More specifically, what type of primes may facilitate lying behavior?

To respond to the first two questions, we have reviewed prime-related literature. Nearly three decades ago, scholars began to notice the influence of prime, although the concept of priming remained limited. Becker (1968) first examined a number of criminal cases and discovered that negative constructs (e.g., immoral, dishonest) are associated with negative behaviors such as defamation, deception and imposture. Becker interprets these behaviors as the consequences of constructed influence, and he analyses such influence through economic principles. Becker’s findings may not directly prove the existence of prime–lying nexus, but findings do imply that constructs may act as a drive to activate the subsequent behavior. More recently, Cohen and colleagues conducted a series of studies, analyzing the influence of identity and business context on behavior. Cohn et al. (2013) have found that priming bankers with their professional identity increases their dishonest behavior. This is because that bankers generally believe that other finance professionals are doing the same things (dishonesty behavior), everyone in the finance industry manipulates figures, so there is no ground for them to act as a red herring. Interestingly, Cohn & Maréchal (2015) also have discovered a similar increase in dishonest behaviors in prisoners by activating their criminal identity. Simply put, prior studies have implied that, when a certain type of prime is activated, dishonest behavior such as lying occurs in the absence of conscious intervention.

To respond to the third question, we have focused on the prime which is caused by politicians (hereafter, referred to as. *politician-priming*). *Politician-priming* is particularly important nowadays, especially when many political leaders are blamed for unethical behavior such as lying and corruption. Generally speaking, people feel that politicians are liars. The first example is: David Cameron (2010–2016 Prime Minister, UK) was first voted for his honesty and upright characters, but he was being investigated for scandals later on, i.e., he first lied to the general public about his innocence of no involvement in offshore activities, but he soon admitted that he had received profit (£30,000) from his father’s offshore investment fund (Independent, 2016). Another example is French citizens distrust their political leaders; only 25% of the national population trust their government and 87% regard their politicians with distrust and disgust (Barometer of Political Trust, 2013). The third example is more general that the public tends to describe politicians as untrustworthy and dishonest, e.g., politicians look after their personal interests more than their public interests (Independent, 2016).

In view of what has preceded, when people perceive politicians as liars, can such perception lead to lying? Perhaps, we are unable to answer this question directly, but Dijksterhuis and van Knippenberg (2000) have provided some valuable insights, which may help to answer the question. They have discovered that self-focus makes alternative behavioral cues salient, which then leads to active inhibition of the stereotype and its effects on behavior. They state that *automatic effects* of environment (e.g., society events, stereotype, work ethos) can guide people’s behavior in the absence of conscious intervention, but the effects may be compromised sometimes. Following this logic, when people regard politicians as liars, their opinion has a potential to affect their subsequent behavior, such as lying. More specifically, politicians often rationalize their lying behavior, so that lying seems to become less guilty and more society-acceptable, e.g., politicians often argue that I lie because there is a good reason behind or I lie to protect national security. Lying has a merit and lying seems not so guilty as long as there is a good reason behind it (Blake, 2013). We are of the view that this rationalization process has provided a good credence to support the relationship between politician-priming and lying; that is, *when people see politicians justifying their lies with a rationale, people may engage in lying (politicians lie so can I)*.

### Research design and hypothesis

Inspired by the prime theories and priming-cognate studies, we have examined the potential prime–lying relationship and proposed an innovative hypothesis that *politicians make people lie via the priming effect*. In this research, we have conducted a series of experiments to examine the hypothesis, using different types of primes. These primes include: politicians, non-politicians (e.g., clergymen), French presidents and French political parties. These primes are genuine and exist in reality; so, the selection of these primes appears sensible and shall contribute to the research ecological validity and interpretation of data analysis (*similar research strategies are adopted in*: Epley and Gilovich 1999; Smeets and Brenner 1995). More specifically, in Experiment 1, the influence of politician-priming and non-politician-priming on lying behavior is compared and analyzed. In Experiment 2—Part A (2A), we aim to further examine the influence of politician-priming; so two presidents of the French Republic are used as primes in the study, including: François Hollande (incumbent at the time of experiment) and Nicolas Sarkozy (preceded). In Experiment 2—Part B (2B), we aim to scrutinize whether politician-priming is individual oriented or party oriented. To sum up, Experiments 1, 2A and 2B aim to jointly analyze the influence of politician-priming from different perspectives, and the findings will not only help to examine the research hypothesis, but also contribute to the knowledge of lying in the absence without conscious intervention.

## Experiment 1

### Sample

We recruited 110 participants who were both employees and part-time students, registered on business programs at a French business school. Participants held a variety of jobs from local public sector workers, retail employees and within SMEs. Participants were randomly assigned to the pre-determined conditions (details of the conditions are clarified later). The mean ages and gender ratio of participants in each condition are shown in Table 1.

**Table 1:** Profile of participants (Experiment 1)

	Baseline (no prime effect)	Non-politician-prime (clergymen-prime effect)	Politician-prime (politician-prime effect)
*N*	41	34	35
Age			
Means	20.43	18.85	20.14
Std. dev	0.10	1.44	0.65
Gender ratio			
Females (%)	68.29	50.00	54.25
Males (%)	31.71	50.00	45.75

### Design and procedure

To examine the influence of politician-priming on lying behavior, we conducted a series of experiments, in which participants played dice games in scenario-based conditions. Scenario-based design often has been adopted for topics in which *real-life* testing would raise ethical concerns from stakeholders including university ethics boards (e.g., Celse et al., 2016; Shalvi, Handgraaf, & De Dreu, 2011). In the case of lying behavior, it would not be desirable to encourage this type of behavior in the workplace and observe any effects. Thus, scenario-based design offers an opportunity to scrutinize the behavior of participants in a quasi-experiment setting whilst removing the ethical issues (Sansone et al. 2004). Following this logic, Experiment 1 crafted three scenario-based conditions. These were:

*baseline*, in which participants received no prime effect;

*politician-prime*, in which participants received politician-prime effect; and finally

*Non-politician-prime*, in which participants received clergy-prime effect.

With respect to the experimental procedure, when participants arrived at the laboratory (experiment site), they were randomly assigned to individual cubicles. Each person was assigned to either *baseline, politician-prime* or *non-politician-prime* condition, i.e., one person stayed in one condition only. Participants then received instruction sheets, subject to their assigned conditions (details of condition manipulation are clarified later). Within each condition, apart from the given instruction sheets, all participants received the same information again from their cubicle monitors and speakers (this design ensured that everyone in the same condition received the same information; the audio- and visual-based information also enhanced the efficacy of instructions, facilitating participants to play dice games).

All participants were told that they would participate in a series of dice games and their performance would be recorded for the purpose of future data analysis and post-experiment rewards, i.e., higher dice readings equate to more monetary rewards (#1 = €1). An administrator was also present throughout the dice games to provide general support to the participants.

During the instruction, five participants were randomly selected to play dice games and read aloud their dice readings. This arrangement not only helped participants to familiarize themselves with dice games, but also ensured participants that their dices were normal, genuine and not cheating-device embedded. After the instruction session, dice games started. Participants were invited to shake the dice in the cup and record the readings on the answer sheet. At this stage, participants only understood that they were expected to play dice games, but they did not know how many rounds of games they played. Specifically, the first two rounds of dice games were pilot exercises only, whereas the third round of game was used for data analysis. The rationale behind the design was to tackle with the bias of end-game effects on data interpretation, so hat the end-game effect could be prevented (Chang, 2012). When the third round of dice games was completed, participants were told that their dice games had finished. They immediately returned their equipment (dice and cup) to the administrator, so that no one could know the outcome of the third round of games. Participants were then requested to leave the laboratory, with a copy of the briefing sheet, revealing the true purpose of the research. Finally, after completing a short questionnaire (details will be clarified later), participants were paid in cash (based on their self-reported dice readings, #1 = €1 … #6 = €6) and thanked for their efforts in the experiment.

### Probability of lying

Across three conditions, participants played a variant of the conventional dice-under-cup paradigm, i.e., a dice was placed inside an opaque cup and there was a peephole lid on the top (see similar design in: Shalvi et al. 2011; Weisel and Shalvi 2015). This design allowed participants to see dice readings through the peephole and only the participants could see the actual readings. Celse et al. (2016) comment that such design has incurred a probability of lying, as participants can choose to *lie* (reporting higher readings to gain higher earnings) and *not lie* (reporting actual readings). This is because the statistical probability of reporting a specific reading (from #1 to #6) is one in six and hence 16.67%. If a specific reading appeared more often, say, the appearance of #6 is higher than the average probability (16.67%), we believe such phenomenon shall be regarded as a sign of lying. Yet, we should not underestimate the possibility that someone may get #6 all the time, so we will evaluate this possibility and discuss its implication later.

### Manipulation of prime conditions

Experiment 1 crafted different scenarios to activate different primes, allowing researchers to observe the effect of different primes on lying behavior. Details follow.

*Politician-prime* Participants received politician-prime effect in this condition. Prior to the dice games, participants received the following instruction:

“Please use the provided sheet of paper to describe the characteristics of politicians. You are free to express your opinions and comment politicians’ characteristics in your own way. This sheet of paper belongs to you and will not be collected by the administrator, so the anonymity of your views is guaranteed. You are encouraged to write as many characteristics as you can on this sheet of paper. You have five minutes for this task”.

*Non-politician-prime* (*clergymen-prime*) This condition adopted the same instruction as above with one exception: we replaced politicians by clergymen. The rationale included: (1) compared to politicians, he general public tends to describe clergymen more positively (e.g., higher moral standards, less dishonest behaviors); so clergymen seem to be a sensible contrast to politicians (see similar manipulation in: Mazar et al., in which participants were primed with religious/moral reminders and their dishonest behavior then decreased); (2) clergymen are common across societies and different types of religions, so clergymen shall not be regarded as artificial stimuli and cause bias in experiments; and finally (3) the arrangement of non-politician-prime (clergymen-prime in this case) shall help examine the efficacy of prime activation and manipulation: when the prime activation and manipulation is successful, lying occurrence shall be lower in the non-politician-prime condition (compared to the baseline or politician-prime conditions). For the convenience of data analysis and comparison across conditions, non-politician-prime is renamed as clergymen-prime in the following discussion.

*Baseline* Participants received no prime activation in this condition and hence no prime manipulation was needed. *Baseline* condition acted as a control group, aiming to compare the influence of different primes on lying occurrence. For instance, if lying occurrence varies (either increase or decrease) in the *politician-prime* condition, *baseline* condition can help comparison across three conditions.

*Manipulation check* Although prime manipulation and dice games were arranged separately, we examined whether participants connected the two via a post-experiment questionnaire and a debriefing session. Results showed that no participant made such connection and so the chance of causal bias was slim, indicating a successful manipulation.

## Findings of Experiment 1

### Reported outcomes of the three conditions

**Fig. 1:**
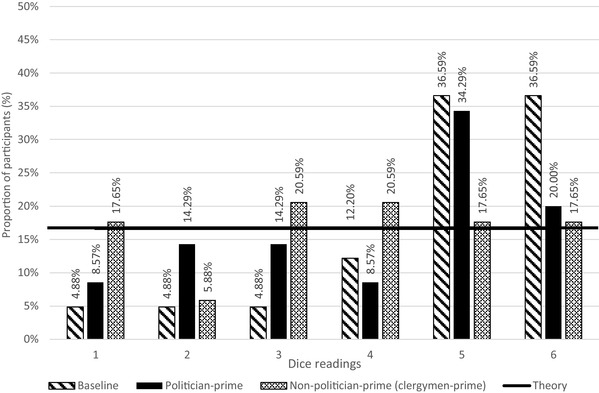
Distribution of dice readings in Experiment 1

We inspected any signs of lying behavior by examining the distribution of reported outcomes (dice readings) of the three conditions, including: *baseline, politician-prime* and *clergymen-prime* (see Fig. 1 for details). As a dice has six sides, the probability of reporting a specific number (from #1 to #6) is one in six, i.e., 16.67%. Interestingly, the probability of reporting #6 is 36.59% in baseline, 20.00% in politician-prime and 17.65% in clergymen-prime. These figures are higher than the probability (16.67%), implying signs of lying, i.e., participants may have lied in reporting their dice readings.

In baseline and politician-prime, readings of lower numbers on the dice (i.e., #1 and #2) were reported less frequently (less than probability), whereas higher readings (i.e., #5 and #6) were reported more frequently (more than probability). The lower readings in baseline and politician-prime appeared consistently less than the probability (16.67%), including: #1 (baseline 4.88%, politician-prime 8.57%) and #2 (baseline 4.88%, politician-prime 14.29%). Conversely, higher readings in baseline and politician-prime conditions appeared consistently more than the probability (16.67%), including: #5 (baseline 36.59%, politician-prime 34.29%) and #6 (baseline 36.59%, politician-prime 20.00%).

The phenomenon that bigger dice readings (i.e., #5 and #6) were reported more often may be explained by two assumptions: (1). participants lied in reporting their dice readings and, (2) participants were indeed lucky to roll out bigger numbers. To further examine these assumptions, further analyses were adopted to examine the distribution of reported readings. To be exact, significant differences across readings #1 to #6 were identified at baseline (Kolmogorov–Smirnov, *D* = 0.532, *p* = 0.001, *p* < 0.01), indicating that the distribution of the reported readings deviated from uniform distribution (note: uniform distribution is based on a principle that the readings from #1 to #6 are equally distributed). Significant differences across readings #1 to #6 were also identified in politician-prime (Kolmogorov–Smirnov, *D* = 0.343, *p* = 0.001, *p* < 0.01).

This phenomenon suggested that participants in baseline and politician-prime lied, i.e., they reported bigger numbers (rather than being lucky to roll out bigger numbers). Interestingly, no significant differences were observed in clergymen-prime (Kolmogorov–Smirnov, *D* = 0.176, *p* = 0.240, *p* > 0.1), indicating that participants in clergymen-prime did not lie in their dice readings. An alternative interpretation is: when participants in clergymen-prime reported bigger readings, their readings were genuine and hence they were not lying. This finding has echoed a prior study that moral reminders act as a lying barrier, refraining dishonest behavior (Mazar et al. 2008). To sum, the results above jointly support the view that participants lied in the *baseline* and *politician-prime* and that participants were less likely to lie in *clergymen-prime*.

### Evaluation of prime effect

We examined the prime effect by comparing the distribution of dice readings across conditions. The distribution of dice readings between baseline and politician-prime was not significantly different (Kolmogorov–Smirnov, *D* = 0.225, *p* = 0.245, *p* > 0.1). The distribution of dice readings between politician-prime and clergymen-prime was not significantly different (Kolmogorov–Smirnov, *D* = 0.189, *p* = 0.469, *p* > 0.1). The average rewards were €4.057 in politician-prime and €3.676 in clergymen-prime. Participants in politician-prime (*M* = €4.057, SD = 1.643) did not report significantly higher readings than participants in clergymen-prime (*M* = €3.673, SD = 1.700) (Mann–Whitney, *p* = 0.334, *p* > 0.1).

However, the distribution of dice readings between baseline and clergymen-prime was significantly different (Kolmogorov–Smirnov, *D* = 0.378, *p* = 0.001, *p* < 0.01), indicating that participants lied more in baseline than clergymen-prime. The average rewards were €4.804 in baseline and €3.676 in clergymen-prime. Participants in baseline (*M* = €4.804, SD = 1.382) reported significantly bigger dice readings than participants in clergymen-prime (*M* = €3.673, SD = 1.700) (Mann–Whitney, *p* = 0.002, *p* < 0.01), indicating that lying behavior was lower in clergymen-prime (in comparison to baseline). To sum up, clergymen-prime was successful as it generated a significant effect on participants’ lying behavior. When participants were primed with clergymen identity, they were less likely to report big dice readings and the distribution of readings was also closer to the probability.

### Evaluation of personal characters

At the end of the dice games, participants were invited to rate the subject that they chose in the game. Three questions were instructed to the participants via an anonymous questionnaire survey, and their responses were recorded on a seven-point *Likert* scale. These questions were: “In terms of trust, how would you rate the subject that you chose in the game? (1 = not trustworthy at all, 7 = completely trustworthy)”, “In terms of honesty, how would you rate the subject that you chose in the game? (1 = not honesty at all, 7 = completely honesty)” and “In terms of admiration, how would you rate the subject that you chose in the game?” (1 = I do not admire the subject at all, 7 = I admire the subject completely). Further statistical analysis was carried out and the results were as follows.

*Level of trust* Participants in clergymen-prime (*M* = 4.794, SD = 1.409) and politician-prime (*M* = 2.771, SD = 1.373) showed significant difference in their ratings (Mann–Whitney, *p* = 0.001, *p* < 0.01). These figures indicated that participants in clergymen-prime described their subject with higher level of trust.

*Level of honesty* Participants in clergymen-prime (*M* = 5.470, SD = 1.211) and politician-prime (*M* = 3.285, SD = 1.446) showed significant difference in their ratings (Mann–Whitney, *p* = 0.001, *p* < 0.01). These figures indicated that participants in clergymen-prime described their subject with a higher level of honesty.

*Level of admiration* Participants in clergymen-prime (*M* = 3.764, SD = 1.596) and politician-prime (*M* = 3.285, SD = 1.601) did not show difference in their ratings (Mann–Whitney, *p* = 0.226). Yet, clergymen-prime’s ratings were still higher than the middle point of seven-point *Likert* scale (*M*_diff_ = 0.264), whereas politician-prime’s ratings were lower than the middle point (*M*_diff_ = − 0.215).

To sum up, participants perceived clergymen to be more positive and politicians less positive; for instance, participants rated clergymen with a higher level of trust and honesty (compared to politicians). It is therefore reasonable to argue that the effect of clergymen-prime has triggered a sense of moral reminder (or commandment); so participants are less likely to lie when they report their dice readings. More specifically, except for the participants in clergymen-prime, participants in baseline and politician-prime showed signs of lying. This phenomenon is meaningful in several ways: (1) participants in clergymen-prime showed no sign of lying and hence they were the most honest participants in the dice games. This finding is dovetailed to our research expectations; (2) participants in baseline lied because they were exposed to an opportunity of lying and lying brings them benefits: this finding is coherent with prior studies of lying incentives (Evans and Lee 2013; Bok 1999); (3) participants in politician-prime lied but their lying occurrence was lower than those in baseline: this finding is intrigue as there is no significant difference (of lying occurrence) between politician-prime and baseline, and between politician-prime and clergymen-prime.

As shown in Fig. 1, the lying occurrence in politician-prime is approximately situated between baseline and clergymen-prime (see exact figures of #5 and #6 in Fig. 1). Such result has challenged our understanding of prime effect on lying occurrence, but we hereby propose an assumption to interpret the result: Before rolling the dice, some participants may choose a politician they like (e.g., a honest and moral one) and so the prime effect reinforces their consequent honest behavior, whereas others may choose a politician they dislike (e.g., a dishonest and immoral one) and so the prime effect reinforces dishonesty behavior. Our assumption is plausible in essence and will be further examined in Experiment 2A.

## Experiment 2—part A (2A)

Experiment 1 activated politician-prime and observed its impact on lying behavior. Although the findings are valuable, a limitation is that the identity of politicians is uncertain and uncontrollable, incurring bias(es) in data analysis. For instance, people may not necessarily describe politicians negatively, as the perception and evaluation of politicians may depend upon the selected politicians. To overcome this limitation, Experiment 2A refined the research scope by narrowing the influence of politicians to the individual level. Following this logic, two recent presidents of the French Republic were used as primes. These were: François Hollande (incumbent at the time of experiment; 2012–2017) and Nicolas Sarkozy (preceded; 2007–2012). Emmanuel Macron (incumbent; 2017–2022) was elected after the experiment (May 2017) and hence not considered for the purpose of prime.

The arrangement of president-prime was based on four reasons. (1). Participants are French citizens and native French speakers. They live and work in France and so it is reasonable to assume that both presidents are familiar faces to participants, i.e., both presidents are clear identity subjects to participants. (2). Hollande and Sarkozy belong to different political parties (Socialist Party vs. The Republicans). They are different in their political orientation (left wing vs. right wing), i.e., Hollande and Sarkozy have different political identity and characters from the participants. (3). Nearly 75% of the French citizens distrust their government, especially the presidents, e.g., Sarkozy was accused of lying and failing to take responsibility in a scandal over the funding of his 2012 presidential campaign, whereas Hollande was accused of dodging tax and lying in his relationships with females (Barometer of Political Trust, 2013); and, (4W). The president is the legal representation of public authority, such as the leader of the state. Analyzing president-activated prime helps us to examine the research hypothesis, i.e., whether politicians make people lie.

### Sample

We recruited 72 participants who were both employees and part-time students, registered on business programs at a French business school. These participants did not attend Experiment 1 and hence had no repeat-effect bias. Participants held a variety of jobs from local public sector workers, retail employees and within SMEs. Participants were randomly assigned to the pre-determined conditions (details of the conditions are clarified later). The mean ages and gender ratio of participants in each condition are shown in Table 2.

**Table 2:** Profile of participants (Experiment 2A)

	Sarkozy-prime	Hollande-prime
*N*	36	36
Age		
Means	21.73	20.36
Std. dev	1.23	1.02
Gender ratio		
Females (%)	54.25	38.84
Males (%)	45.75	61.16

### Design and procedure

Experiment 2A adopted Experiment 1’s design and procedure to measure the effect of primes.

### Manipulation of prime conditions

Experiment 2A crafted two conditions to activate different primes, allowing researchers to observe the effect of different primes on lying behavior. Details follow.

*Sarkozy-prime* Participants received Sarkozy-prime effect in this condition. Prior to the dice games, participants received the following instruction:

“Please use the provided sheet of paper to describe the characteristics of President Sarkozy. You are free to express your opinions and comment Sarkozy’ characteristics in your own way. Your opinions and comment should be about Sarkozy. This sheet of paper belongs to you and will not be collected by the administrator, so the anonymity of your views is guaranteed. You are encouraged to write as many characteristics as you can on this sheet of paper. You have five minutes for this task”.

*Hollande-prime* This condition adopted the same instruction as above, but with one exception: Sarkozy was replaced by Hollande.

*Manipulation check* Experiment 2A adopted Experiment 1’s examination method and the results indicated a successful manipulation.

## Findings of experiment 2—part A (2A)

### Reported outcomes of two conditions

**Fig. 2:**
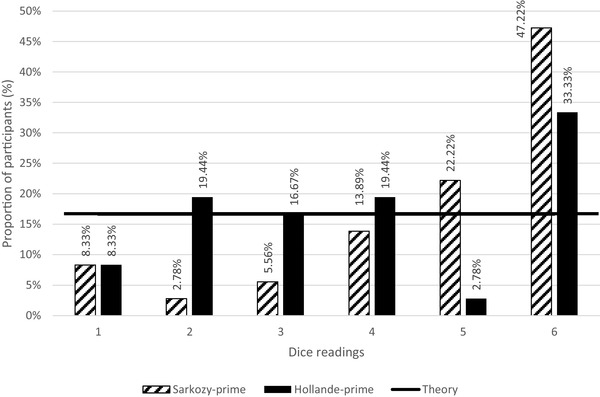
Distribution of dice readings in Experiment 2 (part one)

We inspected any signs of lying behavior by examining the distribution of reported outcomes (dice readings) of the two conditions (Sarkozy-prime vs. Hollande-prime). We discovered significant difference between Sarkozy-prime and Hollande-prime (Kolmogorov–Smirnov, *D* = 0.333, *p* = 0.022, *p* < 0.05). The probability of reporting bigger dice readings was higher in Sarkozy-prime (#5 = 22.22% ; #6 = 47.22%) and lower in Hollande-prime (#5 = 2.78%; #6 = 33.33%). The probability in Sarkozy-prime was higher than that in theory, probably ‘[probability_diff_ (#5) = 5.55%; probability_diff_ (#6) = 30.55%], implying signs of lying, i.e., participants in Sarkozy-prime lied when reporting their dice readings (see Fig. 2 for details).

In Sarkozy-prime, the probability for each dice reading was: #1 (8.33%), #2 (2.78%), #3 (5.56%), #4 (13.89%), #5 (22.22%) and #6 (47.22%). These probability figures showed a general ascending trend from smaller dice readings to bigger readings. In Hollande-prime, such ascending trend did not emerge, as the probability for each reading was: #1 (8.33%), #2 (19.44%), #3 (16.67%), #4 (19.44%), #5 (2.78%) and #6 (33.33%). Further analysis showed that Sarkozy-prime and Hollande-prime showed different dice reading distributions (*χ*^2^ = 13.14, *p* = 0.022, *p* < 0.05), indicating that participants in Sarkozy-prime were more inclined to report bigger dice readings (more lying behavior) whereas participants in Hollande-prime did not show this inclination.

The average dice readings in Sarkozy-prime were 4.805, which were equal to €4.805 in reward. The average dice readings in Hollande-prime were 3.888, which were equal to €3.888 in reward. The average reward in Sarkozy-prime (*M* = €4.805, SD = 1.564) was higher than that of Hollande-prime (*M* = €3.888, SD = 1.789) (Mann–Whitney, *p* = 0.031, *p* < 0.05), indicating that participants in Sarkozy-prime reported big dice readings (i.e., #5 and #6) more often than the participants in Hollande-prime. This phenomenon may be explained by two assumptions: (1) participants lied in reporting their dice readings and, (2) participants were indeed lucky to roll out big numbers.

To further examine these assumptions, further analyses continued. First, significant differences across readings #1 to #6 were identified in Sarkozy-prime (Kolmogorov–Smirnov, *D* = 0.494, *p* = 0.0002, *p* < 0.01), indicating that the distribution of reported readings deviated from uniform distribution. Significant differences across readings #1 to #6 were also identified in Hollande-prime (Kolmogorov–Smirnov, *D* = 0.333, *p* = 0.001, *p* < 0.01). This phenomenon suggested that participants in both Sarkozy-prime and Hollande-prime lied, i.e., they reported bigger numbers (rather than being lucky to roll out bigger numbers). Yet, when comparing the probability of bigger dice readings (#5 and #6) between the two conditions, more signs of lying behavior appeared in Sarkozy-prime [probability (#5) = 22.22%; probability (#6) = 47.22%; average = 34.72%] than in Hollande-prime [probability (#5) = 2.78%; probability (#6) = 33.33%; average = 18.06%].

By comparing the probability of smaller dice readings (#1 and #2) between the two conditions, we found more signs of lying behavior in Sarkozy-prime [probability (#1) = 8.33%; probability (#2) = 2.78%; average = 5.56%] than in Hollande-prime [probability (#1) = 8.33%; probability (#2) = 19.44%; average = 13.89%]. These statistical figures indicated that participants in Sarkozy-prime increasingly reported bigger dice readings to get more rewards, but decreasingly reported smaller readings to avoid losing their rewards (compared to the participants in Hollande-prime). To sum up, participants in Sarkozy-prime reported bigger dice readings than participants in Hollande-prime. They also reported big readings more frequently than their counterparts: namely, Sarkozy-prime leads to more lying behavior, both in gravity (i.e., bigger dice numbers) and frequency (i.e., lying more frequently).

## Experiment 2—part B (2B)

Experiment 2A discovered that Sarkozy-prime led to lying behavior both in gravity and frequency. This finding is valuable, but the mechanism of prime is not clear and hence requires further examination. Participants may evaluate Sarkozy negatively and then associate him with dishonest characteristics, in which Sarkozy-prime activates the tendency of dishonesty behavior (lying of bigger dice readings). Yet, as Sarkozy was the leader of *Union for a Popular Movement* (UMP) and *The Republicans* (note. UMP was founded in 2002 and renamed The Republicans in 2015), it is also reasonable to assume that participants may associate Sarkozy with his political right wing party and therefore the influence of Sarkozy-prime is actually based on the evaluation of *The Republicans*, rather than Sarkozy per se. In view of what preceded, Experiment 2B was conducted to examine the mechanism of Sarkozy-prime and its influence on lying behavior. Specifically, Experiment 2B aimed to scrutinize whether politician-priming was individual oriented or party oriented.

### Sample

Several weeks after Experiment 2A, the same group of participants attended Experiment 2B, by which participants were exposed to a new prime (i.e., Republicans-prime). Participants were randomly assigned into pre-determined conditions (details of the conditions are clarified later). The mean ages and gender ratio of participants are shown in Table 3.

**Table 3:** Profile of participants (Experiment 2B)

	Sarkozy-prime	Republicans-prime (right wing party)
*N*	36	36
Age		
Means	21.53	20.50
Std. dev	1.05	0.23
Gender ratio		
Females (%)	58.83	36.11
Males (%)	41.17	63.89

### Design and procedure

Experiment 2B adopted Experiment 1’s design and procedure to measure the effect of primes.

### Manipulation of prime conditions

Experiment 2B observed how different primes (Sarkozy-prime vs. Republicans-prime) affected lying behavior. 72 participants were randomly assigned to either Sarkozy-prime condition or Republicans-prime condition; so there were 36 participants in each condition. As participants were exposed to Sarkozy-prime in Experiment 2A, researchers decided not to arrange Sarkozy-prime in the manipulation, to reduce the influence of repeat-effect bias in Experiment 2B. For the same reason, participants who were assigned to the Sarkozy-prime condition were requested to leave the experiment and thanked for their participation.

Participants who were assigned to the Republicans-prime condition continued in the experiment. Prior to the dice games, participants received the following instruction:

“Please use the provided sheet of paper to describe the characteristics of The Republicans Party. You are free to express your opinions and comment in your own way. Your opinions and comment should be about The Republicans Party. This sheet of paper belongs to you and will not be collected by the administrator, so the anonymity of your views is guaranteed. You are encouraged to write as many characteristics as you can on this sheet of paper. You have five minutes for this task”.

#### Manipulation check

Experiment 2B adopted Experiment 1’s examination method and the results indicated a successful manipulation.

## Findings of Experiment 2—part B (2B)

### Reported outcomes of the two conditions

We inspected any signs of lying behavior by examining the distribution of reported outcomes (dice readings) in the two conditions, i.e., *Sarkozy-prime* (findings from Experiment 2A) and *Republicans-prime* (findings from Experiment 2B). We discovered no difference between Sarkozy-prime and Republicans-prime (Kolmogorov–Smirnov, *D* = 0.184, *p* = 0.510, *p* > 0.100). However, the probability of reporting bigger dice readings was high in Republicans-prime (#6 = 33.33%) and even higher in Sarkozy-prime (#5 = 22.22% ; #6 = 47.22%). The probability of reporting smaller dice readings was low in Republicans-prime (#1 = 13.89%; #2 = 5.56%) and even lower in Sarkozy-prime (#1 = 8.33%; #2 = 2.78%).

**Fig. 3:**
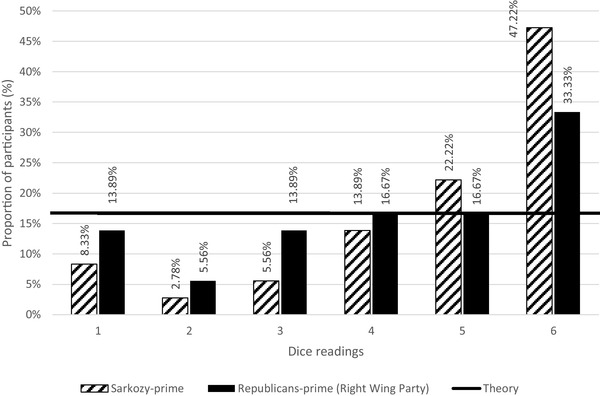
Distribution of dice readings in Experiment 2 (part two)

These statistical figures jointly have suggested the signs of lying, i.e., participants from both conditions lied in reporting their dice readings (see Fig. 3 for details). Specifically, in Sarkozy-prime, the probability for each dice reading was: #1 (8.33%), #2 (2.78%), #3 (5.56%), #4 (13.89%), #5 (22.22%) and #6 (47.22%). These probability figures showed a general ascending trend from smaller dice readings to bigger readings. Significant differences across readings #1 to #6 were identified in Sarkozy-prime (Kolmogorov–Smirnov, *D* = 0.494, *p* = 0.0002, *p* < 0.01), indicating that the distribution of the reported readings deviated from uniform distribution. In Republicans-prime, such ascending trend also emerged, as the probability for each reading was: #1 (13.89%), #2 (5.56%), #3 (13.89%), #4 (16.67%), #5 (16.67%) and #6 (33.33%). Differences across readings #1 to #6 were also identified in Republicans-prime (Kolmogorov–Smirnov, *D* = 0.333, *p* = 0.001, *p* < 0.01), indicating that the distribution of the reported readings deviated from uniform distribution. Namely, the phenomenon that bigger dice readings were reported in both conditions (Sarkozy-prime and Republican-prime) was due to lying behaviors rather than being lucky to roll out bigger numbers. Participants from Sarkozy-prime and Republicans-prime both showed lying behavior.

### Evaluation of Sarkozy-prime and Republicans-prime

The average dice readings in Sarkozy-prime were 4.805, which was equal to €4.805 in rewards. The average dice readings in Republicans-prime were 4.166, which was equal to €4.166 in rewards. The average rewards between Republicans-prime (*M* = €4.166, SD = 1.769) and Sarkozy-prime (*M* = €4.805, SD = 1.564) were not significant (Mann–Whitney, *p* = 0.334, *p* > 0.100). Yet, when comparing the probability of bigger dice readings (#5 and #6) between the two conditions, we can see more signs of lying behavior in Sarkozy-prime [probability (#5) = 22.22%; probability (#6) = 47.22%; average = 34.72%) than in Republicans-prime (probability (#5) = 16.67%; probability (#6) = 33.33%; average = 25.00%). By comparing the probability of smaller dice readings (#1 and #2) between the two conditions, we found more signs of lying behavior in Sarkozy-prime (probability (#1) = 8.33%; probability (#2) = 2.78%; average = 5.56%) than in Republicans-prime [probability (#1) = 13.89%; probability (#2) = 5.56%; average = 9.73%]. Participants in Sarkozy-prime increasingly reported bigger dice readings to get more rewards; simultaneously, they decreasingly reported smaller readings to avoid losing their rewards (compared to the participants in Republicans-prime). To sum up, Republicans-prime yielded an impact on more lying behavior, and Sarkozy-prime made such impact even stronger.

## Discussion and conclusion

Do politicians make people lie? Do presidents exert an influence on the tendency of lying? Inspired by the prime theories, we proposed an innovative hypothesis that people who perceive politicians to be dishonest are likely to lie themselves. In this research, we conducted a series of experiments to examine the influence of politicians on lying occurrence via different primes. Experiment 1 discovered that participants with non-politician-prime (i.e., clergymen-prime) were less likely to lie, and that participants in clergymen-prime described their subject with a higher level of trust and honesty. In contrast, participants in politician-prime described their subject with a lower level of trust and honesty. Experiment 2A discovered that, compared to Hollande-prime, Sarkozy-prime led to lying behavior both in gravity (i.e., bigger lies) and frequency (i.e., lying more frequently). Experiment 2B discovered that Republicans-prime yielded an impact on more lying behavior, and Sarkozy-prime made such impact even stronger. Overall, findings have helped to explain that lying can be triggered by external influencers such as presidents and politicians, i.e., politicians do make people lie. Three experiments jointly have provided an alternative perspective to the conventional view of lying as conscious-driven or self-protection behavior and hence our findings are valuable. The implications of our research findings and suggestions for lying intervention are discussed further.

### Contribution to the knowledge of lying behavior

Lying seems to be a ubiquitous behavior across societies and Serota et al. (2010) describe lying as a social ability, which is essential for polite interaction and self-preservation. In a similar vein, other scholars regard lying as an intentional behavior, lying is the outcome of pay-off evaluation/calculation, and lying brings interests to the liar (e.g., Beck, 1968; Bok 1999; DePaulo 2004). Following this logic, one may argue that lying is a conscious and intentional behavior, and liars lie because they want to lie. Following the prime theories, however, we suggest a different view that lying is not necessarily a conscious and intentional behavior and lying is possible to occur in the absence of conscious intervention. We have learned from literature review that human behavior can be initiated automatically by presenting relevant situational cues (primes) with the mediation of non-conscious perceptual or evaluative processes (Meyer and Schvaneveldt 1971). In this research, we therefore examined the concept of prime and its potential influence on lying behavior. We also examined the impact of primes on lying occurrence by manipulating different types of primes. In sum, research hypotheses were in accordance with our expectations and overall findings have contributed to the knowledge of lying behavior, especially in the field of unintentional lying.

As mentioned in prior studies that lying is often classified as a conscious behavior, our findings have provided an alternative perspective that lying may occur unintentionally and liars may not be aware of the fact that they are lying. In Experiment 1, politicians-prime and clergymen-prime exerted difference in inducing lying behavior (supported by statistics), in which clergymen-prime helped to refrain lying occurrence but such refraining effect on lying did not exist in politician-prime. This refraining effect has also echoed the evaluation of subjects in prime-conditions, e.g., participants in clergymen-prime described their subject with a higher level of trust and honesty, whereas participants in politicians-prime described their subject with a lower level of trust and honesty. In Experiment 2A, although participants from both Sarkozy-prime and Hollande-prime lied, statistical analysis has suggested that Sarkozy-prime led to lying behavior both in gravity (i.e., bigger dice numbers) and frequency (i.e., lying more frequently). In Experiment 2B, although participants from Sarkozy-prime and Republicans-prime both lied, participant in Sarkozy-prime reported bigger dice readings more often. Statistical analysis has suggested that Sarkozy-prime effect may originate from Sarkozy, rather than his political party. Across the three experiments, all findings have made it clear that when people perceive politicians to be dishonest, they are likely to lie. For the same reason, we are of the view that politicians do have a chance to make people lie.

### Limitations and future research

Although we claim that politicians may make people lie through unconscious priming effect, our claim should be interpreted with caution. In reality, people may perceive the same politician differently and hence their evaluation varies, implying that the influence of politician-prime on lying may not be universal. The possibility of conscious lying behavior still exists, as participants may deliberate to get more rewards (more lying) in the experiments. Future studies are encouraged to develop more complex research design, so that both conscious and unconscious lying behavior could be examined simultaneously.

Due to the limited research fund, only two experiments with fixed number of participants were conducted. The limitation in participant recruitment may affect the effect size and undermine the legitimacy of the statistical power. Future studies may extend our research scope and enlarge the participant pool to achieve more robust and reliable research findings. Next, the current research defines unconscious lying as lying without individual awareness. This definition may receive criticism if unconscious lying were actually triggered by self-serving inclination; for instance, lying may be triggered by personal values and the liar may not necessarily be aware of the lying behavior. Future studies may further examine this type of inclination and its implication to lying behaviour.

In terms of prime manipulation, Experiment 2A adopted two French presidents as primes. President is often the head of state (e.g. supreme leader or representative of the entire country) and hence has a great distance to general public (compared to local councillors). Yet, whether distance affects priming effect (e.g., president-prime vs. councillor-prime) was not measured in the research; so future studies are encouraged to examine this distance factor further.

In terms of political-prime, we did not measure voting orientation and its relevance to the level of support for Sarkozy or other candidates. A hindsight is perhaps voting orientation is a better predictor to lying behavior, e.g., if people perceive Sarkozy to be trustworthy and definitely vote for Sarkozy, they would be less likely to lie when they report their dice readings, and vice versa. In addition, we have claimed that Sarkozy-prime leads to lying behavior both in gravity and frequency and its prime effect may originate from Sarkozy, rather than his political party. Although our claims are supported by statistics, we shall not underestimate the possibility that there might be other factor(s) which explain why Sarkozy-prime has a salient effect in inducing lying behavior. Probably, we just have not found it yet.

### Practical implication to lying intervention

Our research has affirmed the importance of prime on behavior and, specifically, we discovered the potential influence of politician-prime on lying behavior. Although mental representation has been found to be crucial in human behavior, scholars seem to have mixed views about why and how mental presentations work. For example, thinking about elderly makes people walk slower (Bargh et al. 1996), thinking about religious statements makes people more likely to be honest (Mazar et al. 2008), and thinking about finance-related job makes people more likely to be dishonest if they were bankers (Cohen et al., 2014). Our research may not explain how and why mental presentation works in the examples above. However, our research has found statistical evidence to explain why and how lying can be triggered by external influencers such as presidents and politicians, and this provides an alternative perspective to the current view of lying as personal interest-driven or self-protection behavior. For the same reason, government leaders and organizational managers should be aware of the risk that they may become the source of lying behavior. One solution to rectify this is that governments, councils and general organizations should craft higher moral standards and HR policies for the recruitment and appraisal of senior and executive members.

There are numerous studies explaining the positive impact of leaders on organizational performance, e.g., they motivate employees and facilitate teams to work more efficiently (see a full review in: Yukl 2012). Different from prior findings that acclaim the importance of leaders, we argue that leaders may become the origins of negative evaluation (Supported by Experiment 1) and trigger lying behavior (Supported by Experiments 2A and 2B). In practice, organizations can apply any lying intervention polities into their personnel training and management, but such policies probably will not reach the maximum efficacy if the organization leader(s) are perceived and evaluated negatively by the majority of members in the organization. Applying more punitive polices (sanctions) helps to reduce lying occurrence temporarily, but such policies may also lead to other side effect (Celse et al., 2016). Following this logic and inspired by Experiment 1 that clergymen-prime helped to refrain lying occurrence, we hereby suggest organization leaders and business owners to attend corporate social reasonability and business ethics workshops on a regular basis, exposing themselves to enterprise morality and ethics more often. Hopefully, these leaders and owners would benefit from the workshops and then exercise their leadership and management with higher moral and ethical standards. Ultimately, with higher enterprise morality and business ethics in practice, organization leaders and business owners are less likely to be evaluated negatively and become the source of lying behavior.

## Conclusion

This research analyzed whether political leaders make people lie via three priming experiments. We proposed an innovative concept that people who perceive leaders to be dishonest (such as liar) are likely to lie themselves. Experiment 1 discovered that participants with non-politician-prime were less likely to lie (compared to politician-prime). Experiment 2A discovered that, compared to Hollande-prime, Sarkozy-prime led to lying behavior both in gravity (i.e., bigger lies) and frequency (i.e., lying more frequently). Experiment 2B discovered that Republicans-prime yielded an impact on more lying behavior, and Sarkozy-prime made such impact even stronger. The research findings suggest that lying can be triggered by external influencers such as leaders, presidents and politicians in the organizations. Our findings have provided valuable insights into organizational leaders and managers in their personnel management practice, especially in the intervention of lying behavior. Our findings also have offered new insights to explain non-conscious lying behavior.
